# Plasma levels of soluble urokinase-type plasminogen activator receptor (suPAR) and early mortality risk among patients enrolling for antiretroviral treatment in South Africa

**DOI:** 10.1186/1471-2334-7-41

**Published:** 2007-05-17

**Authors:** Stephen D Lawn, Landon Myer, Nonzwakazi Bangani, Monica Vogt, Robin Wood

**Affiliations:** 1 Desmond Tutu HIV Centre, Institute for Infectious Disease and Molecular Medicine, Faculty of Health Sciences, University of Cape Town, Cape Town, South Africa; 2 Clinical Research Unit, Department of Infectious and Tropical Diseases, London School of Hygiene and Tropical Medicine, London, UK; 3 Infectious Diseases Epidemiology Unit, School of Public Health and Family Medicine, Faculty of Health Sciences, University of Cape Town, South Africa; 4 Department of Epidemiology, Mailman School of Public Health, Columbia University, New York, USA

## Abstract

**Background:**

Serum concentrations of soluble urokinase-type plasminogen activator receptor (suPAR) have a strong independent association with HIV-1-related mortality. The practical utility of plasma suPAR in assessing short-term all-cause mortality risk was evaluated in patients with advanced immunodeficiency enrolling in an antiretroviral treatment (ART) programme in South Africa.

**Methods:**

An enzyme-linked immunosorbent assay (ELISA) was used to measure plasma concentrations of suPAR in patients at the time of enrolment to the ART programme. The association between plasma suPAR concentrations, baseline patient characteristics and cohort outcomes after 4 months of ART were determined.

**Results:**

Patients (n = 293, 70% female) had a median age of 33 years and were followed up for a median of 5 months from enrolment. The median CD4 cell count was 47 cells/μl (IQR = 22–72) and 38% of patients had WHO stage 4 disease. 218 (74%) patients remained alive after 4 months of ART; 39 (13%) died and 36 (12%) were lost to the programme for other reasons. Patients who died had significantly higher plasma suPAR concentrations compared to those who either survived (P < 0.01) or left the programme for other reasons (P < 0.043). In multivariate analysis, higher log_10 _suPAR concentrations were significantly associated with lower CD4 cell counts, WHO clinical stage 4 disease and male sex. In multivariate analysis to identify factors associated with death, log_10 _suPAR concentration was the most strongly associated variable (P < 0.001). However, examination of sensitivity and specificity characteristics using receiver operating characteristic (ROC) analysis revealed that results from this assay did not have a discriminatory cut-point to provide clinically useful information.

**Conclusion:**

Plasma suPAR concentration was the strongest independent predictor of short-term mortality risk among patients with advanced immunodeficiency enrolling in this ART programme. However, lack of a discriminatory threshold did not permit this marker to be used to triage patients according to short-term mortality risk.

## Background

In 2005 an estimated 25.8 million adults and children in sub-Saharan Africa were living with HIV/AIDS; 4.7 million were in immediate need of antiretroviral treatment (ART) and 2.4 million died [1,2]. Access to ART in the region is expanding and is estimated to have averted 250,000–350,000 deaths in low- and middle-income countries in 2005 alone [3]. Early pessimism that ART could not be effectively delivered on a large scale using a simplified public health approach has largely proven unfounded, at least in the short term. ART access has been rapidly expanded on a massive scale in Lusaka, Zambia, for example, and on a country-wide scale in Malawi with generally good early clinical outcomes [4,5]. However, an important issue to emerge in ART programmes in resource-limited settings is that, following initiation of ART, early mortality is very high [6].

In cohorts in sub-Saharan Africa, on-treatment mortality during the first 12 months of ART ranges between 7% and 26% and predominantly occurs within the initial months of ART [4,7-11]. A report from a South African cohort also found that mortality occurring in the one month period while patients were preparing for ART accounted for 67% of early in-programme deaths [7]. This suggests that even short delays in starting ART may be associated with considerable mortality risk. Mortality is also likely to accrue as a result of delays within the health system upstream of ART programmes [12]. Additional delays may arise due to the practical constraints imposed by the sheer numbers of patients seeking to start treatment in some settings and this may require some form of patient prioritisation.

Delays in starting ART must be minimised for those at high risk of death. Identification of a simple laboratory assay that provides prognostic information beyond that provided by blood CD4 cell count might provide a means of identifying those at highest mortality risk. Such a triage tool might facilitate prioritisation of patients, permitting more rapid initiation of ART and higher intensity medical follow-up in those at greatest risk of death. Low blood haemoglobin and increased serum concentration of B_2_-microglobulin, for example, are each independently associated with mortality [4,10,13]. However, the additional prognostic value of these parameters is somewhat limited.

The plasma concentration of soluble urokinase-type plasminogen activator receptor (suPAR, CD87) is a strong independent predictor of mortality in untreated patients with HIV-1 infection [14,15] and levels decrease in parallel with the response to ART [16]. Plasma concentrations of this immune marker can be quickly and inexpensively measured using a simple enzyme-linked immunosorbent assay (ELISA), which requires much less sophisticated laboratory infrastructure than that needed for CD4 cell count or plasma viral load measurement. Such an assay might therefore be potentially useful in resource-limited settings, especially as the assay could potentially be further simplified to a clinic-based point of care test and could use urine rather than serum [17]. The practical utility of measurements of this immune marker, however, has not been adequately evaluated within clinical HIV services.

We have previously described a community-based ART programme in Cape Town, South Africa, in which mortality rates, risk factors and causes have been reported in detail [7,11,18,19]. In this study we examined the prognostic utility of plasma suPAR concentrations among HIV-infected patients with advanced immunodeficiency and high risk of mortality.

## Methods

### Antiretroviral treatment programme

The ART service based at the Gugulethu Community Health Centre, in Cape Town, South Africa has previously been described [20-22]. The district it serves has a predominantly African population of over 300,000, the vast majority of whom live in conditions of low socioeconomic status. In 2003 the antenatal HIV seroprevalence was 28%. Patients are referred from primary care HIV clinics in the community to the ART programme. Treatment criteria are based on the WHO 2002 recommendations [23], which include those with a prior AIDS diagnosis (WHO stage 4 disease) or a blood CD4 cell count <200 cells/μl.

The mean time between enrolment of a patient in the service and initiation of ART is approximately one month, permitting thorough evaluation of patients (including screening for tuberculosis) and preparation for treatment as described previously [7,11,20,21]. Blood samples are routinely drawn on all patients at a standard clinic visit during this period to measure CD4 cell counts by flow cytometry (FACSCount™, Becton Dickinson, Franklin Lakes, NJ, USA) and plasma viral load using Versant™ HIV-1 RNA 3.0 branched chain DNA assay (Bayer HealthCare, Leverkusen, Germany). Excess EDTA plasma is stored at ≤ -80°C.

First-line ART, comprised of stavudine, lamivudine plus a non-nucleoside reverse transcriptase inhibitor (efavirenz or nevirapine), was supplied free of charge. Treatment adherence and viral load suppression <400 copies/ml in this cohort both exceed 90% at all 16 weekly follow-up time-points [20]. All patients with CD4 counts <200 cells/μl received prophylaxis with daily cotrimoxazole or dapsone as an alternative but isoniazid prophylaxis was not used. In addition to scheduled clinic appointments at 4, 8, and 16 weeks and 16-weekly thereafter, patients had open access to the clinic for medical problems.

Assignment of each patient to a community-based therapeutic counsellor promotes high levels of treatment compliance and also high levels of data-completeness so that final outcomes are known for the vast majority of patients. A proportion of patients did not initiate treatment for a variety of reasons other than death and such individuals were deferred from the programme and follow-up was censored at the time they exited the programme. Principal causes of death in this cohort have been previously been described in detail and include wasting syndrome, acute sepsis, tuberculosis, malignancy and immune reconstitution disease (mainly associated with cryptococcal meningitis) [7,19,24].

Structured clinical records were maintained on all patients screened on entry to the ART programme and this information was transferred on a weekly basis to a computer database. All patients enrolling in studies at this ART clinic provide written informed consent and this study was approved by the Research Ethics Committee of the University of Cape Town.

### Plasma suPAR concentrations

Total plasma suPAR concentrations were measured at a single time-point in plasma samples that had been stored at the time of programme enrolment. In order to assess this prognostic marker a patient population with high mortality risk, we selected all patients who enrolled into the programme between September 2002 and February 2005 and who had a CD4 cell count ≤100 cells/μl.

Plasma suPAR concentrations were measured using a commercially available enzyme-linked immunosorbent assay (ELISA) (suPARnostic,™ ViroGates, Lyngby, Denmark) following the manufacturer's instructions. This is a simple double monoclonal antibody sandwich assay that measures total suPAR, including both full-length and cleaved forms of the receptor. In brief, a standard control curve (range 0.6 – 19.3 ng/ml), positive control, and test samples were incubated in duplicates in a 96-well plate pre-coated with anti-suPAR antibody. Following further incubation with a secondary peroxidase-conjugated antibody, the assay was developed by addition of a tetramethylbenzidine (TMB) chromogenic substrate. The reaction was terminated by addition of sulphuric acid and absorbance at 450 nm was determined using a microtitre plate reader. The linear standard curve was used to determine concentrations in positive control and test samples. Samples with concentrations exceeding the highest standard (19.3 ng/ml) were reanalysed using a further 5-fold sample dilution.

### Data analysis

Outcomes for all patients were determined from the time of entry to the programme until completion of 4 months ART or exit from the programme due to death or other causes. Data were analysed using STATA Version 9.0 (College Station, Texas, USA) and GraphPad Prism 4.0 (GraphPad Software Inc. San Diego, CA, USA). As the frequency distribution of values was highly right-skewed, the suPaR values were log_10_-transformed for bivariate analyses (based on the Mann Whitney U or Kruskall Wallis tests to compare medians) and multivariate modelling. We constructed multiple linear regression models to predict suPAR values at programme enrolment, and used Cox's proportional hazards to examine the associations between suPAR concentrations and the relative hazard of mortality. For the latter set of models, time at risk was calculated from entry into the programme to the first of death, transfer out of the programme, loss to-follow-up, or the 4 month follow-up visit on ART. Receiver operating characteristic (ROC) curves were constructed to examine the effect of different cut-points of log_10 _suPAR values on the sensitivity and specificity of predicting mortality during the study period. Confidence intervals for the area under the ROC curves were calculated using the method described by Bamber [25].

## Results

### Patient baseline characteristics and follow-up

Of patients who enrolled into the ART programme between September 2002 and February 2005, 354 had a blood CD4 cell count ≤100 cells/μl. Of these, a stored plasma sample was available for measurement of suPAR concentration in 293 (83%) patients and these were included in the study. These patients had a median age of 33 years and approximately two-thirds were female (Table 1). 89% had either WHO stage 3 or 4 disease. A total of 43% of patients had either an active diagnosis of TB prior to initiation of ART or developed symptoms during the first 4 months of ART that were due to confirmed incident TB.

**Table 1:** Patient characteristics (n = 293)

Age median (IQR), years	33(29–38)
Female sex	205(70)
Median (IQR) CD4 cell count	47(22–72)
WHO Stage	
1/2	32(11)
3	150(51)
4	111(38)
Viral load > 10^5 ^copies/ml	149(51)
Tuberculosis,	
Present at enrolment	80(27)
Developed during ART	47(16)
Median (IQR) duration	
follow up (days)	145(139–173)
Outcome	
Alive on ART	218(74)
Deaths pre or during ART	39(13)
Non-death losses	36(12)

Baseline characteristics and outcome of patients enrolling in the antiretroviral treatment (ART) programme who were included in the analysis. Unless otherwise stated, values are numbers (%) of patients.

Twenty-four (8%) patients did not start ART due to death before starting treatment (n = 13) or other reasons (n = 11), including treatment refusal, loss to follow-up or access to treatment at another clinic. A total of 269 (92%) patients did start ART. Of these, 218 (82%) were retained within the programme and were alive after 16 weeks of treatment. Twenty six (9.7%) patients died within the first 16 weeks of treatment. Other programme losses during ART included 15 (5.6%) patients who moved out of area and 10 (3.7%) who were lost to follow-up. The median (IQR) total period of follow-up was 145 days (139–173) and was comprised of a median pre-ART period of 34 days (28–56) and a median of 112 days (112–119) on ART.

### Plasma suPAR concentrations

Detectable levels of suPAR were measured in plasma samples from all 293 patients. The standard curves for each run were linear (mean r^2 ^= 0.995; SD = 0.004) and all positive control readings were consistent with the expected value. The median suPAR concentration in the patient plasma samples was 4.05 ng/ml (IQR, 3.03–5.77; range 1.14–52.4 ng/ml) or 0.607 log_10 _ng/ml (IQR = 0.481–0.761).

Associations between baseline patient characteristics and log_10 _suPAR concentrations were examined (Table 2). In unadjusted analyses, low baseline CD4 cell counts and WHO stage 4 disease were strongly associated with higher log_10 _plasma concentrations of suPAR. There was also a trend towards patients with active TB diagnoses having higher suPAR concentrations. When the cases of tuberculosis were subdivided into those with active TB at the time of the blood sample and those who developed later tuberculosis during ART, neither group was significantly associated with suPAR concentration. Other baseline characteristics, however, were not significantly associated with suPAR concentrations. In adjusted analyses, the association with CD4 cell count and WHO stage remained, but female sex was also found to be significantly associated with lower suPAR concentrations (Table 2).

**Table 2:** 

Patient characteristic	Unadjusted association			Adjusted association		
		
	Mean change log suPAR per unit increase in variable	95% CI	P value	Mean change log suPAR per unit increase in variable	95%CI	P value
Age (years)	-0,0001	-0.0038, 0.0035	0.944	0.0018	-0.0020, 0.0056	0.353
Female	-0.0362	-0.0950, 0.0226	0.227	-0.0615	-0.1228, -0.0002	0.049
CD4 cell count (cells/μl)	-0.0014	-0.0026, -0.0005	0.003	-0.0012	-0.0022, -0.0029	0.011
Log_10 _viral load (copies/ml)	0.0328	-0.0149, 0.0806	0.177	0.0219	-0.0253, 0.0691	0.361
WHO stage 1,2,3 versus 4	0.0691	0.0140, 0.1243	0.014	0.0566	-0.0003, 0.1136	0.051
Tuberculosis (all cases)	0.0482	-0.0087, 0.1051	0.097	0.0380	-0.0187, 0.0947	0.189

Crude associations between baseline patient characteristics and log10 plasma suPAR concentrations (ng/ml) measured at baseline.

### Patient outcomes and suPAR concentrations

To examine the association between log_10 _serum suPAR concentrations and clinical outcome, patients were divided into three groups according to final outcome: (i) received ART and alive after 4 months treatment (n = 218, 74%); (ii) died during the analysis period (n = 39, 13%); or (iii) other programme losses (n = 36, 12%). Among those who died, 13 died before starting ART and 26 died during the first 4 months of ART.

**Figure 1:**
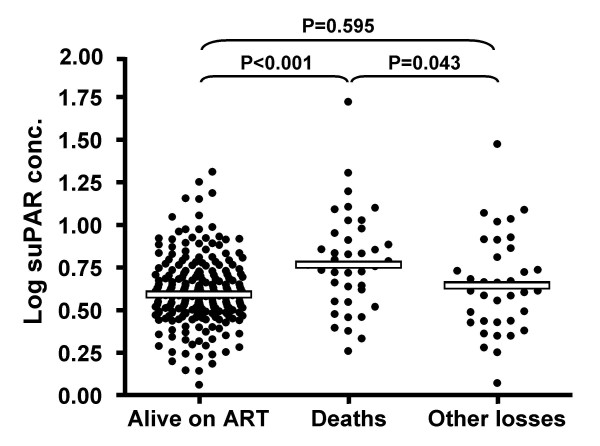
Log_10 _suPAR concentrations among patients (n = 293) enrolling in an antiretroviral treatment (ART) programme. Individual data points represent individual patients who were either receiving ART and alive after completing 4 months of treatment (n = 218), had died before starting ART or during the first 16 weeks of treatment (n = 39), or were lost to the programme (n = 36). Group median values are shown by a bar.

The median log suPAR concentration among patients who died (0.761 ng/ml, IQR = 0.548–0.949 ng/ml) was significantly greater than that of patients who remained alive on ART (median = 0.583 ng/ml, IQR = 0.479–0.715 ng/ml) or who were lost to the programme for reasons other than death (median = 0.609 ng/ml ng/ml; IQR = 0.429–0.833 ng/ml) (Figure 1). However, concentrations did not differ significantly comparing those who remained alive with non-death losses. Thus, higher suPAR concentrations were strongly associated with mortality. However, median suPAR concentrations were almost identical comparing those who died pre-ART with those who died during the first 4 months of ART (5.92 and 5.93 ng/ml, respectively) and suPAR concentrations were not associated with causes of death (data not shown).

### Predictors of mortality

We next examined predictors of mortality using multivariate analysis. In an initial model that included baseline patient characteristics but excluded suPAR concentration, only baseline CD4 cell count was significantly associated with mortality. There was also a strong trend towards an association with WHO clinical stage (Table 3a). However, when the log concentration of plasma suPAR was included in the model, the strength of the associations with CD4 cell count and WHO stage were diminished and log suPAR concentration was found to be the strongest independent predictor of mortality (Table 3).

**Table 3:** 

**Characteristic**	**(a) Hazard ratio**	**95% CI**	**P value**	**(b) Hazard ratio**	**95% CI**	**P value**
Age	1.00	0.96–1.05	0.844	1.00	0.96–1.04	0.985
Male	1.30	0.65–2.60	0.464	1.68	0.84–3.39	0.142
Baseline CD4 cell count	0.99	0.97–1.00	0.028	0.99	0.98–1.00	0.059
Log_10 _Viral load (copies/ml)	0.94	0.50–1.78	0.848	0.85	0.45–1.61	0.612
WHO stage 4	1.91	0.98–3.73	0.059	1.62	0.83–3.16	0.162
Tuberculosis (all cases)	1.13	0.59–2.17	0.710	0.95	0.49–1.86	0.883
Log suPAR concentration ng/ml	-	-	-	9.96	2.78–35.7	<0.001

Cox proportional hazards model showing the association between baseline characteristics of patients (n = 293) and subsequent death before starting antiretroviral treatment (ART) or during the first 16 weeks ART. The first model (a) shows associations with baseline patient characteristics whereas the second model (b) also includes plasma log suPAR concentration.

**Figure 2:**
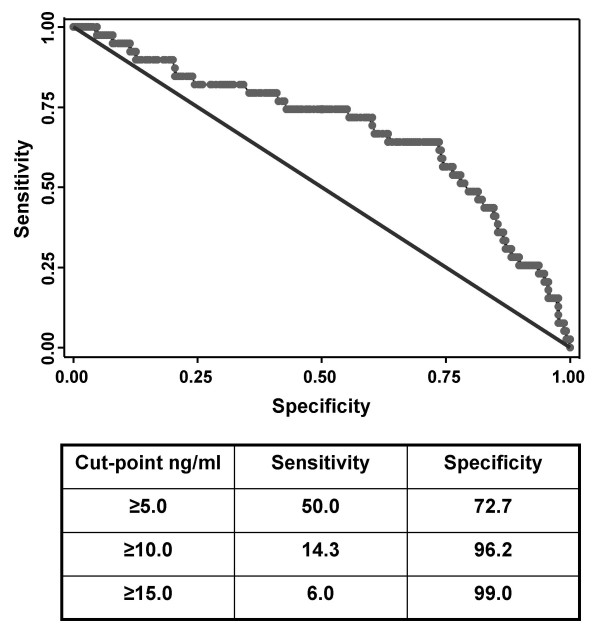
Receiver operating characteristic (ROC) curve analysis showing the utility of plasma suPAR concentration as a prognostic test predicting death up to the first 4 months of ART. The area under the curve is 0.681.

Having demonstrated that plasma suPAR concentration was strongly associated with mortality at a group level, we next examined the utility of this as a prognostic test to be applied to individual patients. To do this, receiver operating characteristic (ROC) curve analysis was done (Figure 2). The area under the ROC curve was moderate (0.681; 95% CI 0.582–0.781) and yet a clear cut-point that maximised the ability of suPAR concentration to distinguish between patients who died or survived was lacking. Using cut-points of ≥5.0 ng/ml, ≥10.0 ng/ml or ≥15 ng/ml, the corresponding sensitivity and specificity values showed that none of these cut-offs provided sufficient sensitivity and specificity that would be clinically useful in this patient population (Figure 2).

## Discussion

In this study we examined the utility of plasma suPAR concentrations for predicting all-cause mortality among HIV-infected patients with advanced immunodeficiency early in an ART programme in South Africa. Our observations add to previous findings from Europe that plasma suPAR is an independent predictor of mortality in untreated cohorts of HIV-infected persons [14,15]. We also found that mortality was more strongly associated with suPAR concentration than with any other risk factor including CD4 cell count and WHO clinical stage. However, despite these strong associations, there was no useful cut-point in the distribution of suPAR concentrations that could discriminate adequately between groups with higher and lower mortality risk. Thus, the marker was unable to serve as a useful adjunctive tool to assess short-term mortality risk in this patient population.

SuPAR is a component of the plasminogen activation system, which comprises urokinase-type plasminogen activator (uPA) and its receptor (uPAR) [26]. uPAR is expressed on a variety of different immune cells and vascular endothelial cells. It is involved in the recruitment of leukocytes from the circulation to extravascular sites of inflammation through regulatory effects on pericellular proteolysis, cell adhesion, chemotaxis, and signal transduction [26,27]. suPAR is generated by either proteolytic cleavage or shedding from cells. Serum concentrations are increased and prognostic in a variety of inflammatory and neoplastic disease states, including HIV infection, tuberculosis, sepsis, rheumatoid arthritis and certain malignancies and suPAR concentrations reflect prognosis in these disease states [14,15,17,28-30].

In European cohorts of HIV-infected patients plasma suPAR concentrations were higher among those with more advanced clinical stages of disease [14,15]. This is likely to reflect the fact that systemic immune activation increases with HIV disease progression and is central to the pathogenesis of the infection [31]. In this study we also found that higher suPAR concentrations were significantly associated with advanced WHO stage of disease and lower blood CD4 cell counts. Absolute levels reported in this patient population are consistent with those found in previous studies [14,15]. High suPAR concentrations were also found to be weakly associated with male sex. This has not previously been reported although it is interesting that male sex was an independent risk factor for early mortality in several African ART cohorts [4,11,32]. Although tuberculosis has been found to be associated with increased supAR levels [28] there was no such association in this study. This may reflect the fact that those who were free of tuberculosis may have had a high frequency of other opportunistic infections, which also increase suPAR concentrations, thereby masking any association with TB.

The association between high suPAR concentrations and mortality was highly statistically significant both at a group level (Figure 1) and in multivariate analysis (Table 3). However, the distributions of suPAR concentrations in those who survived and those who died were very broadly overlapping (Figure 1). This is likely to reflect the fact that suPAR is a non-specific inflammatory marker with a continuous distribution. As a result, using ROC analysis it was not possible to identify a useful cut-point that provided adequate sensitivity and specificity to enable the assay to be used to discriminate those at highest risk of death. This assay could therefore not be used to triage patients according to mortality risk as they enrolled into this ART programme.

A strength of this study is that we studied patients in a community-based service that is likely to be representative of other public sector ART clinics elsewhere in sub-Saharan Africa. Moreover, a cohort rather than case control design reduced potential for patient selection bias. Completeness of outcome data in this cohort was high due to active community-based follow-up of patients using peer counsellors. Some patients who were lost to the programme for reasons other than death may have subsequently died. However, these patients were categorised separately in the group analysis and their median suPAR concentration did not differ from that of the group who remained alive. Previous studies have only examined associations between plasma suPAR concentrations and prognosis in HIV-infected patients at a group level. This is the first study to evaluate the practical clinical utility of this marker in a clinical setting.

The study assessed short-term mortality risk over a median of 5 months from enrolment. An important reason for this is that we have previously reported that deaths in this period are strongly associated with baseline patients characteristics [11] whereas deaths occurring beyond this period are only associated with response to ART. Previous studies of the prognostic value of suPAR concentration have reported on much longer durations of follow-up of 3 years [15] and 5 years [14] in natural history cohorts. Plasma suPAR concentrations may have greater prognostic value in assessing long-term mortality risk when assessed in patients with less advanced immunodeficiency. However, short-term mortality risk is of greatest relevance to clinicians making clinical decisions and for developing treatment guidelines [33,34].

Only patients with advanced immunodeficiency were included and the restricted cohort composition may have diminished the association between mortality and risk factors such as CD4 cell count. This would also explain the lack of association with plasma viral load [35]. Despite the cohort composition, the association between mortality and plasma suPAR concentrations was nevertheless very strong at a group level. The data from this study are relevant to patients with advanced disease who are already eligible for ART and cannot be generalised to those with earlier disease. Whether this assay can provide useful prognostic information relevant to when ART should be started needs to be assessed in future studies. This will depend on whether the distribution of suPAR concentrations in patients with earlier stages of disease has a discriminatory threshold.

In conclusion, at a group level plasma suPAR concentrations are strongly predictive of mortality among HIV-infected patients in sub-Saharan Africa, confirming the findings of previous studies in Europe. However, the lack of a cut-point that provides adequate sensitivity and specificity to predict mortality in this patient population prevented this assay from providing clinically useful prognostic information for individual patient management.

## Competing interests

The author(s) declare that they have no competing interests.

## Authors' contributions

SDL, RW and LM designed the study and analysis. NB and MV did the laboratory work. SDL wrote the manuscript with critical input from RW and LM. All authors read and approved the final manuscript

## Pre-publication history

The pre-publication history for this paper can be accessed here:


